# Aberrant functional connectivity and activity in Parkinson’s disease and comorbidity with depression based on radiomic analysis

**DOI:** 10.1002/brb3.2103

**Published:** 2021-03-10

**Authors:** Xulian Zhang, Xuan Cao, Chen Xue, Jingyi Zheng, Shaojun Zhang, Qingling Huang, Weiguo Liu

**Affiliations:** ^1^ Department of Radiology Nanjing Medical University Affiliated Nanjing Brain Hospital Nanjing China; ^2^ Institute of Brain Functional Imaging Nanjing Medical University Nanjing China; ^3^ Division of Statistics and Data Science Department of Mathematical Sciences University of Cincinnati Cincinnati USA; ^4^ Department of Mathematics and Statistics Auburn University Auburn USA; ^5^ Department of Statistics University of Florida Gainesville USA; ^6^ Department of Neurology Nanjing Medical University Affiliated Nanjing Brain Hospital Nanjing China

**Keywords:** depression, machine learning, Parkinson's disease, radiomics

## Abstract

**Introduction:**

The current diagnosis of Parkinson's disease (PD) comorbidity with depression (DPD) largely depends on clinical evaluation. However, the modality may tend to lack precision in detecting PD with depression. A radiomic approach that combines functional connectivity and activity with clinical scores has the potential to achieve accurate and differential diagnosis between PD and DPD.

**Methods:**

In this study, we aimed to employ the radiomic approach to extract large‐scale features of functional connectivity and activity for differentiating among DPD, PD with no depression (NDPD), and healthy controls (HC). We extracted 6,557 features of five types from all subjects including clinical characteristics, resting‐state functional connectivity (RSFC), amplitude of low‐frequency fluctuation (ALFF), regional homogeneity (ReHo), and voxel‐mirrored homotopic connectivity (VMHC). Lasso, random forest, and support vector machine (SVM) were implemented for feature selection and dimension reduction based on the training sets, and the prediction performance for different methods in the testing sets was compared.

**Results:**

The results showed that nineteen features were selected for the group of DPD versus HC, 34 features were selected for the group of NDPD versus HC, and 17 features were retained for the group of DPD versus NDPD. In the testing sets, Lasso prediction achieved the accuracies of 0.95, 0.96, and 0.85 for distinguishing between DPD and HC, NDPD and HC, and DPD and NDPD, respectively. Random forest achieved the accuracies of 0.90, 0.82, and 0.90 for distinguishing between DPD and HC, NDPD and HC, and DPD and NDPD, respectively, while SVM yielded the accuracies of 1, 0.86 and 0.65 for distinguishing between DPD and HC, NDPD and HC, and DPD and NDPD, respectively.

**Conclusions:**

By identifying aberrant functional connectivity and activity as potential biomarkers, the radiomic approach facilitates a deeper understanding and provides new insights into the pathophysiology of DPD to support the clinical diagnosis with high prediction accuracy.

## Graphical abstract

By identifying aberrant functional connectivity and activity as potential biomarkers, the radiomics approach facilitates a deeper understanding and provides new insights into the pathophysiology of DPD to support the clinical diagnosis with high prediction accuracy.

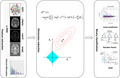

## 1 INTRODUCTION

Depression is a frequent psychiatric symptom of Parkinson's disease (PD) and one of the earliest prodromal comorbidities that can significantly impact quality of life (Chagas et al., 2013). Nonmotor features including depression can appear in the earliest phase of the disease even before clinical motor impairment (Lix et al., 2010; Shearer et al., 2012; Tibar et al., 2018). The efficacy of medications and psychotherapies for treating depression in PD patients remains limited (Abós et al., 2017). Hence, advances in timely detection and concerted management of PD comorbidity with depression (DPD) become urgent. Motor symptoms were easily detected than nonmotor symptoms using the present diagnostic tools (Picillo et al. 2017). According to the Unified Parkinson's Disease Rating Scale (UPDRS), over half DPD patients were not recognized by neurologists (Lachner et al. 2017), while the incidence of PD with depression was already substantially elevated recently (Kay et al., 2018). Clearly, physician recognition and current understanding for comorbidity of depression in PD are not enough.

Although knowledge of the neural and pathophysiologic mechanisms of DPD progression remains limited, many researchers are devoted to conduct research trying to understand the inner working mechanisms and discovering biomarkers of DPD. Clinical intervention is urgent around the early therapeutic windows (Tibar et al., 2018; Vu et al., 2012). Multimodal neuroimaging methods such as functional magnetic resonance imaging (MRI) and electroencephalography have aided the diagnosis of PD. Resting‐state functional MRI (rs‐fMRI) can provide more information on functional connections to assess the correlations among different networks. An intra‐ and internetwork functional connectivity study in DPD demonstrated aberrant functional connectivity (FC) in left frontoparietal network, basal ganglia network, salience network, and default‐mode network (DMN) (Wei et al., 2017). Meanwhile, these connectivity anomalies were correlated with the depression severity in DPD. This may indicate the mechanism of progressive deterioration and compensation for integrative neural models in DPD (Wei et al., 2017; Zhu et al. 2016). Structural MRI has also received research attention because of its stability and repeatability (Jacob et al., 2019; Remes et al., 2011). Diffusion tensor imaging can discover microstructural changes in the brain white matter. Previous studies found abnormal white matter fiber characteristic (mainly located in the right arcuate fasciculus and bilateral middle cerebellar peduncles) in prodromal early stage of PD (Sanjari Moghaddam et al., 2019). Another microstructure difference was located in the bilateral white matter fiber of the mediodorsal thalamic regions between the DPD and NDPD groups, but the sample size was relatively small and the clinical score only included the Hamilton depression rating scale (HAMD)(Li et al., 2010).

In recent years, machine learning has been recognized as a promising and powerful algorithm method for prediction and medical diagnosis. Studies have been conducted to obtain voxel‐based morphological biomarkers of PD by using machine learning such as support vector machine (SVM) or principal component analysis (PCA) that allowed individual differential diagnosis of PD (Lix et al., 2010; Palumbo et al., 2014; Salvatore et al., 2014). Another method (Peng et al., 2017; Peran et al., 2010) focusing on region of interest (ROI) has also been implemented where some specific regions of the brain such as gray matter and hippocampal volume were extracted based on prior knowledge regarding their effects on brain functionality and memory.

Recent progress in digital medical image analysis allows us to develop a novel feature extraction method called radiomics which converts large amounts of medical imaging characteristics into high‐dimensional mineable data pool to build a predictive and descriptive model. The method has been applied to some neuropsychiatric diseases such as autism, schizophrenia, and Alzheimer disease (Feng et al., 2019; Salvatore et al., 2019). These findings demonstrate the validity of these radiomic approaches in improving the classification accuracy and discovering discriminative features that can reveal pathological information. A radiomic study on quantitative susceptibility mapping (QSM) achieved good performance for predicting PD (Xiao et al., 2019). The combination of radiomics features and convolutional neural networks (CNN) can increase the diagnostic accuracy (Ortiz et al., 2019). Other radiomic analysis focusing on longitudinal SPECT images and T2‐weighted MRI can also enhance the prediction accuracy of PD (Liu et al., 2020; Rahmim et al., 2017). A radiomic study based on PET/CT images extracted high‐order features and trained a SVM model to classify PD and HC subjects, and the results demonstrated that the radiomic method combined with SVM could distinguish PD from HC (Wu et al., 2019). Cao et al. leveraging rs‐fMRI radiomic features showed that machine learning methods including Lasso and SVM could significantly improve diagnostic accuracy of PD (Cao et al., 2020).

In the present study, we aimed to build and validate a radiomic method that can facilitate the individual diagnosis of patients with PD and the development of DPD by extracting whole‐brain functional connectivity and activity using the radiomic approach. The proposed method can also identify brain regions of interest with aberrant functional activity between DPD and PD that were relevant to the disease onset, which may contribute to the early diagnosis and treatment for clinical practice.

## 2 MATERIALS AND METHODS

(Data acquisition and preprocessing procedures have all been applied in our published issue, Cao, et al., Front Neurosci. 2020; 14:751. 10.3389/fnins.2020.00751).

This prospective study was approved by the institutional review board and followed the ethical guidelines of the Declaration of Helsinki, and written informed consent was acquired from each subject before inclusion.

### 2.1 Participates and clinical evaluation

We used the same imaging data from the same recruited subjects as in our previously published issue (Cao et al., 2020). The only difference is that we further stratify the PD patients into two groups of DPD and NDPD to examine the aberrant functional connectivity and activity in DPD and to build machine learning models for predicting DPD and NDPD. Seventy PD patients including 21 DPD and 49 NDPD subjects were recruited, along with 50 matched healthy controls. The details regarding the diagnostic criteria and clinical evaluation of the NDPD and DPD groups are provided in Data S1.

**FIGURE 1:**
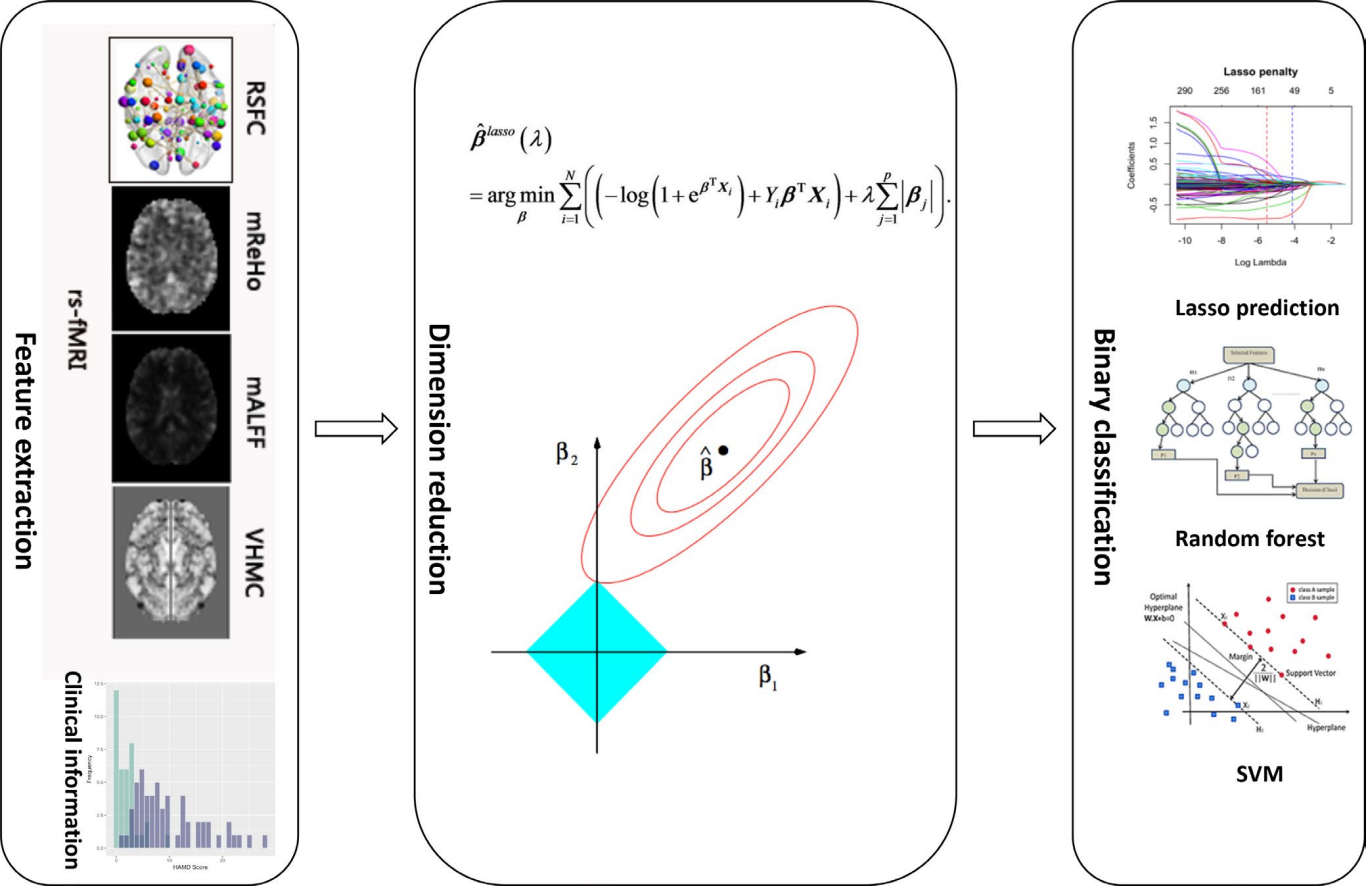
Flowchart of the study. We extracted the 6,557 metrics after the rs‐fMRI images preprocessed. Then, Lasso regression was carried out to reduce the number of features. Last, Lasso prediction, random forest, and SVM were used to differentiate between different categories of subjects

**2.2–2.5** Image data acquisition, preprocessing, extraction of radiomic features including regional homogeneity (ReHo), amplitude of low‐frequency fluctuation (ALFF) and voxel‐mirrored homotopic connectivity (VMHC), resting‐state functional connectivity (RSFC), feature selection, and model validation are provided in Data S2. The flowchart of this study is shown in Figure 1.

## 3 RESULTS

### 3.1 Differences in clinical characteristics

Clinical information from three groups was displayed in Table 1. No significant difference was observed among the three groups regarding age, gender, education, and MMSE score, while significant difference in HAMD score was detected among three groups. In particular, for the DPD group, the HAMD scores (20.2 ± 4.6) were significantly higher than those for other two groups (the same data from our aforementioned published study were used).

**TABLE 1:** Clinical and demographic data evaluation of DPD, NDPD, and HC

Characteristics	DPD (*n* = 21)	nDPD (*n* = 49)	HC (*n* = 50)	Test statistics	*p* value
Sex (M/F)	9/12	26/23	24/26	0.409	>.05^a^
Age (year)	58.1 ± 7.5	57.8 ± 7.0	57.8 ± 5.5	0.021	>.05^b^
Education (year)	11.0 ± 3.1	11.8 ± 3.3	11.7 ± 4.8	0.689	>.05^c^
MMSE	28.7 ± 1.1	28.6 ± 1.7	29.0 ± 2.3	0.585	>.05^d^
HAMD	20.2 ± 4.6	6.9 ± 3.1	2.2 ± 2.3	243.2 (*p* < .05)	<.016^e‐g^ < .016^e‐g^ < .016^e‐g^

^a^ The *p* value for gender distribution by Fisher's exact test.

^b^ The *p* value for age by multivariate analysis of variance (MANOVA).

^c^ The *p* value for education by MANOVA.

^d^ The *F* test statistic and the *p* value for MMSE scores by MANOVA.

^e‐g^ The *p* values for HAMD scores by paired‐samples *t* test with Bonferroni correction for further comparison between three groups.

### 3.2 Feature selection

**FIGURE 2:**
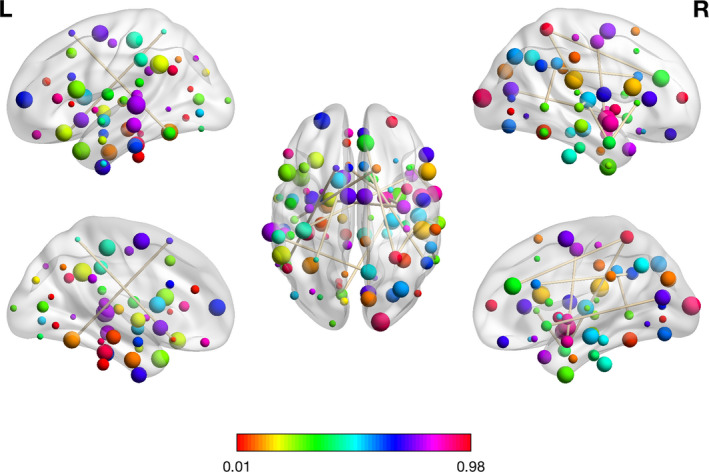
The visualization plot of the selected 16 RSFC features for the first classification between DPD and HC using the BrainNet Viewer (Xia et al., 2013)

For the first classification of DPD versus (versus) HC, 19 features including (HAMD, 2 mALFFs, and 16 RSFCs) were retained for binary classification. The 16 RSFCs and corresponding brain regions using HOA template were presented in Table 2. The most aberrant brain regions of RSFCs included DMN, executive control network (ECN), visual network (VIN), affective network (AN), sensorimotor network (SMN), and short‐term memory (STM) network (Figure 2). The other two mALFF features were located at the left precentral gyrus and the left planum polare. In Table 3, we reported the statistical characteristics of these features resulting from the dimension reduction step and illustrated the difference in these selected features between DPD and HC. The decreasing or increasing trend of these features between DPD and HC can also be discovered in Table 3.

**TABLE 2:** 16 RSFC features and the related brain regions indexed in the HOA template for differentiating DPD from HC

ID	HOA number	Brain region A	Network	HOA number	Brain region B	Network
1	5	Superior Frontal Gyrus.L	DMN	31	Inferior Temporal Gyrus, temporooccipital.L	Other region
2	6	Superior Frontal Gyrus. R	DMN	89	Heschl's Gyrus.L	AUN
3	14	Precentral Gyrus. R	SMN	109	Left Amygdala	DMN
4	14	Precentral Gyrus.R	SMN	110	Right Amygdala	DMN
5	16	Temporal Pole.R	AN	47	Intracalcarine Cortex.L	VIN
6	16	Temporal Pole.R	AN	85	Parietal Operculum Cortex.L	SMN
7	20	Superior Temporal Gyrus, posterior.R	DMN	90	Heschl's Gyrus.R	AUN
8	22	Middle Temporal Gyrus, anterior.R	DMN	36	Superior Parietal Lobule.R(SPL)	VIN
9	22	Middle Temporal Gyrus, anterior.R	DMN	82	Frontal Operculum Cortex.R	VAN
10	26	Middle Temporal Gyrus, temporooccipital.R	FNs	40	Supramarginal Gyrus, posterior.R	STM
11	35	Superior Parietal Lobule.L(SPL)	VIN	53	Subcallosal Cortex.L	Other region
12	36	Superior Parietal Lobule.R(SPL)	VIN	56	Paracingulate Gyrus.R	ECN
13	37	Supramarginal Gyrus, anterior.L	STM	62	Precuneus Cortex.R	DMN
14	42	Angular Gyrus.R	DMN	58	Cingulate Gyrus, anterior.R	SN
15	57	Cingulate Gyrus, anterior.L	SN	110	Right Amygdala	DMN
16	94	Supracalcarine Cortex.R	VIN	112	Right Accumbens	Other region

**TABLE 3:** The mean, standard deviation (*SD*) and *p* value for all 19 selected features in the training sets for the group of DPD versus HC

ID	Features	DPD (mean ± *SD*)	HC (mean ± *SD*)	*p* value
1	Superior Frontal Gyrus.L‐Inferior Temporal Gyrus, temporooccipital.L	0.3854 ± 0.3630	0.1885 ± 0.2118	0
2	Superior Frontal Gyrus.R‐Heschl's Gyrus.L	0.2827 ± 0.3072	0.5094 ± 0.2967	.0606
3	Precentral Gyrus.R‐Left Amygdala	−0.1771 ± 0.3208	0.0576 ± 0.2736	.0314
4	Precentral Gyrus.R‐Right Amygdala	−0.2144 ± 0.2458	−0.0180 ± 0.2247	.0015
5	Temporal Pole.R‐Intracalcarine Cortex.L	0.5120 ± 0.2035	0.2924 ± 0.2869	0
6	Temporal Pole.R‐Parietal Operculum Cortex.L	0.2573 ± 0.1805	−0.1046 ± 0.3396	.0051
7	Superior Temporal Gyrus, posterior.R‐Heschl's Gyrus.R	0.1219 ± 0.2061	−0.1426 ± 0.2739	.1274
8	Middle Temporal Gyrus, anterior.R‐Superior Parietal Lobule.R	−0.0564 ± 0.1229	0.0885 ± 0.2423	.3779
9	Middle Temporal Gyrus, anterior.R‐Frontal Operculum Cortex.R	0.0773 ± 0.1620	−0.1025 ± 0.1783	.1568
10	Middle Temporal Gyrus, temporooccipital.R‐Supramarginal Gyrus, posterior.R	0.0909 ± 0.3364	−0.1273 ± 0.2680	.2875
11	Superior Parietal Lobule.L(SPL)‐Subcallosal Cortex.L	0.0690 ± 0.2970	−0.1440 ± 0.3210	.2622
12	Superior Parietal Lobule.R(SPL)‐Paracingulate Gyrus.R	−0.0626 ± 0.2409	−0.1500 ± 0.2254	.3921
13	Supramarginal Gyrus, anterior.L‐Precuneus Cortex.R	0.1038 ± 0.2217	−0.0251 ± 0.2207	.0950
14	Angular Gyrus.R‐Cingulate Gyrus, anterior.R	−0.0276 ± 0.2388	−0.2765 ± 0.2116	.1589
15	Cingulate Gyrus, anterior.L‐Right Amygdala	0.2119 ± 0.2191	−0.0252 ± 0.2398	.0020
16	Supracalcarine Cortex.R‐Right Accumbens	−0.0057 ± 0.2229	0.2186 ± 0.2920	.0512
17	mALFF of Left Precentral Gyrus	0.8066 ± 0.0847	0.9223 ± 0.1261	.0007
18	mALFF of Left Planum Polare	1.4770 ± 0.2962	1.3442 ± 0.2449	0
19	HAMD Score	20.5385 ± 4.0541	2.3143 ± 2.2198	0

**FIGURE 3:**
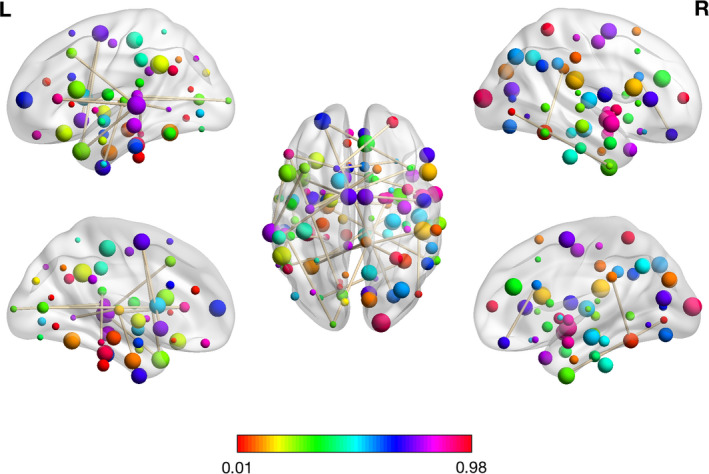
The visualization plot of the selected 30 RSFCs for the second classification: NDPD versus HC

For the second classification, NDPD versus HC, 34 features including (30 RSFCs, HAMD, 1 mALFF, 1ReHo, and 1VHMC) remained for binary classification. The 30 RSFCs and the corresponding brain regions using HOA template were presented in Table 4. The most aberrant brain regions of RSFCs were located in DMN, VIN, AN, SMN, automatic urban network (AUN), ventral attention network (VAN), ECN, salience network (SN), and basal ganglia (BGN) (Figure 3). The other three radiomic features were mALFF of the left juxtapositional lobule cortex, mReHo of the left middle temporal gyrus, posterior division, and VMHC of the right temporal fusiform cortex, posterior division. The mean, standard deviation, and *p* value of these 34 features were reported in Table 5.

**TABLE 4:** 30 RSFC features and the related brain regions indexed in the HOA template for differentiating NDPD from HC

ID	HOA number	Brain region A	Network	HOA number	Brain region B	Network
1	1	Frontal Pole.L	Other region	8	Middle Frontal Gyrus.R	DMN
2	1	Frontal Pole.L	Other region	80	Occipital Fusiform Gyrus.R	VIN
3	2	Frontal Pole.R	Other region	5	Superior Frontal Gyrus.L	Other region
4	4	Insular Cortex.R	SN	88	Planum Polare.R	Other region
5	5	Superior Frontal Gyrus.L	DMN	66	Frontal Orbital Cortex.R	Other region
6	6	Superior Frontal Gyrus.R	DMN	37	Supramarginal Gyrus, anterior.L	STM
7	7	Middle Frontal Gyrus.L	DMN	19	Superior Temporal Gyrus, posterior.L	DMN
8	9	Inferior Frontal Gyrus, pars triangularis.L	Other region	19	Superior Temporal Gyrus, posterior.L	DMN
9	9	Inferior Frontal Gyrus, pars triangularis.L	Other region	22	Middle Temporal Gyrus, anterior.R	DMN
10	9	Inferior Frontal Gyrus, pars triangularis.L	Other region	47	Intracalcarine Cortex.L	VIN
11	13	Precentral Gyrus.L	SMN	82	Frontal Operculum Cortex.R	VAN
12	13	Precentral Gyrus.L	SMN	112	Right Accumbens	Other region
13	15	Temporal Pole.L	AN	19	Superior Temporal Gyrus, posterior.L	DMN
14	15	Temporal Pole.L	AN	51	Juxtapositional Lobule Cortex.L	Other region
15	25	Middle Temporal Gyrus, temporooccipital.L	FNs	89	Heschl's Gyrus.L	AUN
16	27	Inferior Temporal Gyrus, anterior.L	DMN	89	Heschl's Gyrus.L	AUN
17	28	Inferior Temporal Gyrus, anterior.R	DMN	46	Lateral Occipital Cortex, inferior.R	VIN
18	28	Inferior Temporal Gyrus, anterior.R	DMN	77	Temporal Occipital Fusiform Cortex.L	VIN
19	31	Inferior Temporal Gyrus, temporooccipital.L	Other region	92	Planum Temporale.R	Other region
20	32	Inferior Temporal Gyrus, temporooccipital.R	Other region	73	Temporal Fusiform Cortex, anterior.L	VIN
21	50	Frontal Medial Cortex.R	DMN	58	Cingulate Gyrus, anterior.R	SN
22	51	Juxtapositional Lobule Cortex.L	Other region	111	Left Accumbens	Other region
23	60	Cingulate Gyrus, posterior.R	DMN	77	Temporal Occipital Fusiform Cortex.L	VIN
24	60	Cingulate Gyrus, posterior.R	DMN	78	Temporal Occipital Fusiform Cortex.R	VIN
25	68	Parahippocampal Gyrus, anterior.R	DMN	109	Left Amygdala	DMN
26	69	Parahippocampal Gyrus, posterior.L	DMN	85	Parietal Operculum Cortex.L	Other region
27	78	Temporal Occipital Fusiform Cortex.R	VIN	79	Occipital Fusiform Gyrus.L	VIN
28	84	Central Opercular Cortex.R	Other region	103	Left Putamen	BGN
29	91	Planum Temporale.L	Other region	95	Occipital Pole.L	VIN
30	95	Occipital Pole.L	VIN	110	Right Amygdala	DMN

**TABLE 5:** The mean, standard deviation (*SD*), and *p* value for all 34 selected features in the training sets for the group of NDPD versus HC

ID	Features	NDPD (mean ± *SD*)	HC (mean ± *SD*)	*p* value
1	Frontal Pole.L‐Middle Frontal Gyrus.R	0.6287 ± 0.2843	0.4504 ± 0.3413	0
2	Frontal Pole.L‐Occipital Fusiform Gyrus.R	−0.2502 ± 0.2854	−0.4479 ± 0.2228	.0008
3	Frontal Pole.R‐Superior Frontal Gyrus.L	0.1360 ± 0.2409	−0.0666 ± 0.2649	.0033
4	Insular Cortex.R‐Planum Polare.R	0.3087 ± 0.2961	0.1136 ± 0.1984	0
5	Superior Frontal Gyrus.L‐Frontal Orbital Cortex.R	0.1880 ± 0.2725	−0.0482 ± 0.3444	.0009
6	Superior Frontal Gyrus.R‐Supramarginal Gyrus, anterior.L	−0.0965 ± 0.2182	−0.2674 ± 0.2170	.0585
7	Middle Frontal Gyrus.L‐Superior Temporal Gyrus, posterior.L	0.4780 ± 0.2792	0.2907 ± 0.2677	0
8	Inferior Frontal Gyrus, pars triangularis.L‐Superior Temporal Gyrus, posterior.L	−0.0148 ± 0.2314	0.1764 ± 0.2544	.2549
9	Inferior Frontal Gyrus, pars triangularis.L‐Middle Temporal Gyrus, anterior.R	−0.2060 ± 0.2867	0.0127 ± 0.2723	.0001
10	Inferior Frontal Gyrus, pars triangularis.L‐Intracalcarine Cortex.L	−0.1387 ± 0.2596	0.1013 ± 0.2941	.0067
11	Precentral Gyrus.L‐Frontal Operculum Cortex.R	−0.0223 ± 0.2241	−0.2138 ± 0.2068	.2278
12	Precentral Gyrus.L‐Right Accumbens	−0.0666 ± 0.2067	−0.2799 ± 0.2678	.2214
13	Temporal Pole.L‐Superior Temporal Gyrus, posterior.L	−0.2858 ± 0.2406	−0.4705 ± 0.2687	.0002
14	Temporal Pole.L‐Juxtapositional Lobule Cortex.L	−0.1078 ± 0.2802	−0.3625 ± 0.2483	.0976
15	Middle Temporal Gyrus, temporooccipital.L‐Heschl's Gyrus.L	−0.0661 ± 0.2838	−0.2662 ± 0.2636	.2563
16	Inferior Temporal Gyrus, anterior.L‐Heschl's Gyrus.L	0.5797 ± 0.3812	0.2949 ± 0.3318	0
17	Inferior Temporal Gyrus, anterior.R‐Lateral Occipital Cortex, inferior.R	−0.0485 ± 0.1849	0.1095 ± 0.1477	.1335
18	Inferior Temporal Gyrus, anterior.R‐Temporal Occipital Fusiform Cortex.L	0.4466 ± 0.3445	0.1378 ± 0.3444	0
19	Inferior Temporal Gyrus, temporooccipital.L‐Planum Temporale.R	−0.2934 ± 0.2524	−0.1327 ± 0.2430	0
20	Inferior Temporal Gyrus, temporooccipital.R‐Temporal Fusiform Cortex, anterior.L	−0.0219 ± 0.1985	0.1411 ± 0.2267	.1932
21	Frontal Medial Cortex.R‐Cingulate Gyrus, anterior.R	−0.1154 ± 0.2473	0.1462 ± 0.2711	.0200
22	Juxtapositional Lobule Cortex.L‐Left Accumbens	−0.0518 ± 0.2334	0.1499 ± 0.2725	.2808
23	Cingulate Gyrus, posterior.R‐Temporal Occipital Fusiform Cortex.L	0.2302 ± 0.2153	0.0001 ± 0.2783	0
24	Cingulate Gyrus, posterior.R‐Temporal Occipital Fusiform Cortex.R	0.0186 ± 0.2482	0.1740 ± 0.2555	.2036
25	Parahippocampal Gyrus, anterior.R‐Left Amygdala	0.3030 ± 0.2640	0.0721 ± 0.3043	0
26	Parahippocampal Gyrus, posterior.L‐Parietal Operculum Cortex.L	0.1977 ± 0.3141	−0.0680 ± 0.3273	.0008
27	Temporal Occipital Fusiform Cortex.R‐Occipital Fusiform Gyrus.L	−0.0092 ± 0.2235	0.1330 ± 0.2873	.1467
28	Central Opercular Cortex.R‐Left Putamen	0.0590 ± 0.2228	−0.0844 ± 0.2651	.1802
29	Planum Temporale.L‐Occipital Pole.L	0.0788 ± 0.2119	0.2063 ± 0.2260	.0893
30	Occipital Pole.L‐Right Amygdala	0.1418 ± 0.2327	0.0236 ± 0.2305	.0008
31	mALFF of Left Juxtapositional Lobule Cortex	0.9061 ± 0.1372	1.0316 ± 0.1531	0
32	mReHo of Left Middle Temporal Gyrus, Posterior Division	1.0703 ± 0.0994	0.9802 ± 0.0995	0
33	VMHC of Right Temporal Fusiform Cortex, Posterior Division	0.1917 ± 0.0846	0.1331 ± 0.1166	0
34	HAMD score	6.8182 ± 2.9310	2.4545 ± 2.2373	0

**FIGURE 4:**
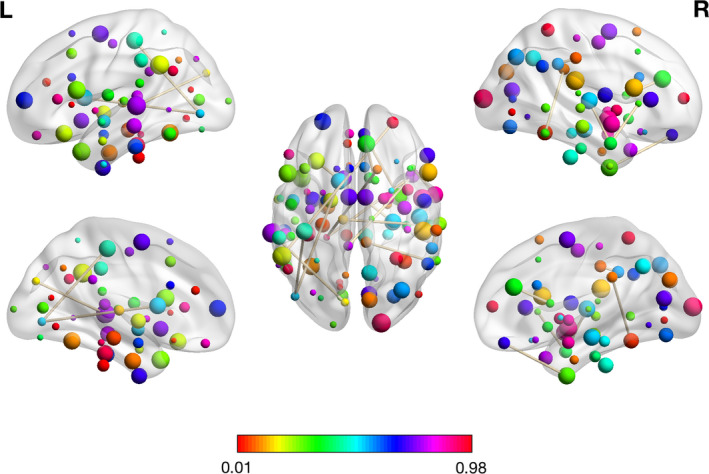
The visualization plot of the selected 15 RSFCs for the third classification: NDPD versus HC

For the third classification, DPD versus NDPD, 17 features including (15 RSFCs, HAMD, and 1 mALFF) were kept for binary classification. The 15 RSFCs and the corresponding brain regions using HOA template were presented in Table 6. The most aberrant networks associated with these RSFCs included the DMN, VIN, STM, AN, BGN, SMN, SN, ECN, VAN, and AUN (Figure 4). The remaining mALFF feature belonged to the region of left subcallosal cortex. In Table 7, we also listed the mean, standard deviation, and *p* value of these 17 radiomic features.

**TABLE 6:** 15 RSFC features and the related brain regions indexed in the HOA template for differentiating DPD from NDPD

ID	HOA number	Brain region A	Network	HOA number	Brain region B	Network
1	2	Frontal Pole.R	Other region	39	Supramarginal Gyrus, posterior.L	STM
2	6	Superior Frontal Gyrus.R	Other region	69	Parahippocampal Gyrus, posterior.L	DMN
3	16	Temporal Pole.R	AN	59	Cingulate Gyrus, posterior.L	DMN
4	16	Temporal Pole.R	AN	69	Parahippocampal Gyrus, posterior.L	DMN
5	19	Superior Temporal Gyrus, posterior.L	DMN	63	Cuneal Cortex.L	Other region
6	20	Superior Temporal Gyrus, posterior.R	DMN	99	Left Thalamus	DMN
7	22	Middle Temporal Gyrus, anterior.R	DMN	82	Frontal Operculum Cortex.R	VAN
8	22	Middle Temporal Gyrus, anterior.R	DMN	90	Heschl's Gyrus.R	AUN
9	32	Inferior Temporal Gyrus, temporooccipital.R	Other region	60	Cingulate Gyrus, posterior.R	DMN
10	33	Postcentral Gyrus,L	SMN	45	Lateral Occipital Cortex, inferior.L	VIN
11	38	Supramarginal Gyrus, anterior.R	STM	40	Supramarginal Gyrus, posterior.R	STM
12	45	Lateral Occipital Cortex, inferior.L	VIN	101	Left Caudate	BGN
13	50	Frontal Medial Cortex.R	SN	74	Temporal Fusiform Cortex, anterior.R	VIN
14	56	Paracingulate Gyrus.R	ECN	102	Right Caudate	BGN
15	65	Frontal Orbital Cortex.L	VIN	108	Right Hippocampus	DMN

**TABLE 7:** The mean, standard deviation (*SD*), and *p* value for all 17 selected features in the training sets for the group of DPD versus NDPD

ID	Features	DPD (mean ± *SD*)	NDPD (mean ± *SD*)	*p* value
1	Frontal Pole.R‐Supramarginal Gyrus, posterior.L	−0.0141 ± 0.2446	−0.1103 ± 0.2266	.1323
2	Superior Frontal Gyrus.R‐Parahippocampal Gyrus, posterior.L	0.0908 ± 0.3179	0.3104 ± 0.3217	.2138
3	Temporal Pole.R‐Cingulate Gyrus, posterior.L	−0.5177 ± 0.1974	−0.2593 ± 0.2495	0
4	Temporal Pole.R‐Parahippocampal Gyrus, posterior.L	0.2872 ± 0.2356	0.0371 ± 0.2611	.0001
5	Superior Temporal Gyrus, posterior.L‐Cuneal Cortex.L	0.7327 ± 0.2467	0.4994 ± 0.1967	0
6	Superior Temporal Gyrus, posterior.R‐Left Thalamus	0.2852 ± 0.2382	0.4485 ± 0.2196	.0194
7	Middle Temporal Gyrus, anterior.R‐Frontal Operculum Cortex.R	0.0727 ± 0.1566	−0.1205 ± 0.2109	.2219
8	Middle Temporal Gyrus, anterior.R‐Heschl's Gyrus.R	0.1312 ± 0.1967	−0.1280 ± 0.2557	.0663
9	Inferior Temporal Gyrus, temporooccipital.R‐Cingulate Gyrus, posterior.R	−0.0184 ± 0.2250	−0.1782 ± 0.2349	.2035
10	Postcentral Gyrus,L‐Lateral Occipital Cortex, inferior.L	0.0495 ± 0.1741	−0.1453 ± 0.2468	.1751
11	Supramarginal Gyrus, anterior.R‐Supramarginal Gyrus, posterior.R	0.0070 ± 0.2991	0.2006 ± 0.2252	.1307
12	Lateral Occipital Cortex, inferior.L‐Left Caudate	0.1091 ± 0.2589	−0.0380 ± 0.1802	.0539
13	Frontal Medial Cortex.R‐Temporal Fusiform Cortex, anterior.R	0.0218 ± 0.2341	−0.1444 ± 0.1815	.2254
14	Paracingulate Gyrus.R‐Right Caudate	−0.2438 ± 0.1765	−0.0544 ± 0.1970	0
15	Frontal Orbital Cortex.L‐Right Hippocampus	−0.0016 ± 0.2287	0.1277 ± 0.2147	.1807
16	mALFF of Left Subcallosal Cortex	0.9220 ± 0.0859	1.0933 ± 0.2287	.0007
17	HAMD Score	20.2143 ± 4.0796	6.8182 ± 3.0460	0

### 3.3 Model fitting

After the screening process, for all three classifications, there were no more than 34 features left, and the ultrahigh dimensional situation was no longer present. Most of the commonly used machine learning methods including Lasso prediction, random forest, and SVM could accommodate this relatively smaller number of variables compared with previous 6,557 features. Hence, we performed the model fitting procedure for categorizing subjects in the training set using Lasso prediction, SVM, and random forest to assess the performance of these methods. After cross‐validation, the results demonstrated that all three methods achieved perfect accuracy and AUC for distinguishing among the three groups in the training sets. The outcome was not surprising as the numbers of coefficients to be estimated were comparable to the sample sizes.

### 3.4 Model validation

**FIGURE 5:**
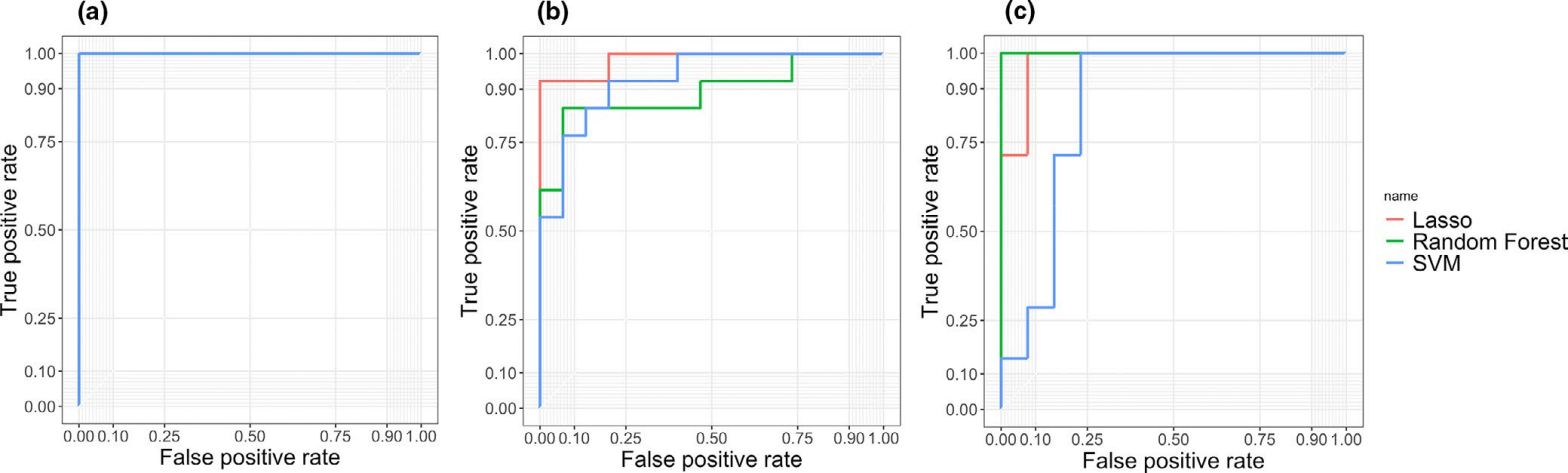
ROC curves displaying the predictive performance of Lasso prediction, SVM, and random forest in the testing sets for three classifications. (a) DPD versus HC; (b) NDPD versus HC; (c) DPD versus NDPD

Although all the methods have achieved superior performance in the training set, the predictive result in the testing set is what really matters. We therefore tested the validity of Lasso, SVM, and random forest by assessing their classified performance in the testing set. The area under the curve (AUC), accuracy, true positive rate (TPR), and true negative rate (TNR) were measured (Table 8). Taking the first categorization: DPD versus HC as an example, TPR measures the proportion of subjects that were corrected identified as DPD by the given procedure within all the DPD subjects. TNR is calculated using the number of HCs detected and divided by all the number of HCs. Accuracy ensures the proportion of true results (including both TP and TN) among the total number of subjects tested (i.e., the sample size of the testing set). The natural cutoff of 0.5 was used to determine whether these subjects should be classified as DPD. To further evaluate the robustness of all three methods, the ROC curves were also plotted by varying thresholding values in Figure 5.

**TABLE 8:** Predictive performance table in the testing set for Lasso prediction, random forest, and SVM

	Accuracy	TPR	TNR	AUC
DPD versus HC				
Lasso	0.95	0.88	1	1
Random forest	0.90	0.75	1	1
SVM	1	1	1	1
NDPD versus HC				
Lasso	0.96	0.92	1	0.98
Random forest	0.82	0.85	0.80	0.89
SVM	0.86	0.85	0.87	0.93
DPD versus NDPD				
Lasso	0.85	0.71	0.92	0.98
Random forest	0.90	0.71	1	1
SVM	0.65	0.29	0.85	0.86

From Table 8 and Figure 5, we can tell that Lasso prediction performed better than random forest and SVM for differentiating NDPD from HC. Random forest outperformed Lasso and SVM for discriminating DPD from NDPD in terms of the overall accuracy. SVM yielded a higher prediction accuracy compared with Lasso and random forest for distinguishing HC from DPD.

## 4 DISCUSSION

By conducting the radiomic analysis, our study presented a comprehensive framework for discovering predictive biomarkers of DPD and for classifying HC, DPD, and NDPD subjects using whole‐brain rs‐fMRI metrics including ReHo, mALFF, VHMC, and RSFC. In our study, PD with depression can be distinguished from HC with a 100% accuracy using SVM, while the accuracies using Lasso and random forest were 0.95 and 0.90, respectively. When comparing DPD and NDPD, the prediction accuracies of Lasso, random forest, and SVM were 0.85, 0.90, and 0.65, respectively. For the group of NDPD and HC, the accuracies of three methods were 0.96, 0.82, and 0.86, respectively. From the aforementioned results, it is not difficult to see that all three methods have achieved high classification accuracy and are also quite robust with respect to varying thresholds based on the AUC values. The method of Lasso achieved the highest accuracy and AUC averaged over three groups. A vast amount of existing literatures also focused on biomarkers for the identification of PD and distinguishing PD from other neurodegenerative diseases. A study using the machine learning method got an accuracy of 0.9 for differentiating PD from progressive supranuclear palsy (PSP) (Salvatore et al., 2014). Another study using SVM correctly identified PD with other comorbidity of tremor‐dominant symptom with an accuracy of 100% using a multimodal algorithm (Cherubini et al., 2014). Consistent with existing methods, the present study also found SVM with a multipredictor model was able to fully discriminate DPD from HC.

As once said by Robert Gilles et al., “Images are more than pictures, they are data” (Gillies et al., 2016) radiomic approaches based on data‐characterization algorithms have been widely applied to disease prediction and diagnosis especially in oncology and genetic fields. A random forest‐based radiomics analysis combining both nonimaging and imaging variables found the longitudinal DAT‐SPECT images significantly improved the prediction accuracy of PD, and exhibited great potentials toward development of effective prognostic biomarkers in PD (Wu et al., 2019). In recent years, a computer‐based technique utilizing CNN (Ortiz et al., 2019; Shinde et al., 2019) to create prognostic and diagnostic biomarkers has been widely adopted and attracted lots of attention. These methods exploited 3D structural MRI and required no prior knowledge on significant regions that might impact the progress of PD. A QSM study showed radiomic features extracted from QSM data had high values in the diagnosis of PD (Xiao et al., 2019). Isosurface‐based features with CNN features enhanced the diagnostic accuracy of PD (Ortiz et al., 2019). In our study, we also discovered discriminative RSFC features in NDPD and DPD, which supported the validity of the radiomic approach. With the emergence of data‐driven approaches, radiomics have been shown to be trustworthy and practically useful to aid PD diagnosis and to reach precision medicine.

The aberrant regions associated with RSFC features contributing to the discrimination of DPD from HC were primarily located in the DMN, ECN, VIN, AN, SMN, and STM. The other two mALFF features were located at the left precentral gyrus and the left planum polare. The disturbed brain regions distinguishing NDPD from HC were located in DMN, VIN, AN, SMN, AUN, VAN, ECN, SN, and BGN. Compared with HC subjects, DPD and NDPD patients shared similar brain network abnormalities mainly in the DMN, VIN, ECN, AN, SMN, STM, ECN, and AUN. The most discriminated regions of RSFCs that differentiated DPD from NDPD were within or across the DMN, VIN, AN, STM, BGN, SMN, SN, ECN, VAN, and AUN. Our results showed that aberrant functional connectivity and activity for DPD were primarily detected within the DMN, VIN, AN, STM, SMN, AUN, VAN, ECN, SN, and BGN. A previous study on FC markers of depression in advanced PD also found RSFC features located in the subcortical, auditory, SMN, VIN, cognitive control, DMN, and cerebellar networks. These networks were significantly relevant to classification and provided preliminary evidence that can characterize DPD patients compared with NDPD (Lin et al., 2019).

DMN plays an important role in self‐referential introspective condition, and disturbances of the DMN have been confirmed in many neurological and psychiatric disorders including PD. A RSFC study in DPD found the increased ALFF in the DMN compared with the NDPD and HCs. Other previous studies also found that the BGN, DMN, LFPN, and SN were involved DPD (Hu, Song, Li, et al., 2015), which facilitated the advancement of more detailed and integrative neural models of DPD (Lin et al., 2019; Wei et al., 2017).

In our study, aberrant functional connectivity and activity of the emotion network and motor network were also identified in DPD patients. Abnormal directional connectivity between motor network and emotion network in DPD has been described in the existing literature. Compared with HC, DPD patients displayed significant gray matter volume abnormality in some limbic and subcortical regions in addition to the unique alterations of directional connectivity from the different brain regions, which may provide differential biomarkers for distinguishing DPD from HC and NDPD (Liang et al., 2016). Rs‐fMRI studies in depression have shown that antidepressant treatment could affect cortical connectivity. The corticolimbic network and amygdala play an important role in the development of DPD, even antidepressant effects also associated with the abnormal hypoconnectivity in DPD (Morgan et al., 2018). Compared to NDPD and HC, DPD showed abnormal functional connectivity in the left amygdala, right amygdala, and the bilateral mediodorsal thalamus. The disturbed connectivity between limbic regions and corticolimbic networks in DPD patients may reflect impaired limbic areas in mood dysregulation of emotion‐related regulatory effect (Hu, Song, Li, et al., 2015).

The group of DPD displayed altered spontaneous brain activity in the frontal, temporal, and limbic regions in our study. Compared with NDPD, DPD exhibited significantly increased regional activity in the superior temporal gyrus, middle temporal gyrus, inferior temporal gyrus, and frontal medial cortex. Decreased RSFC values were detected between superior frontal gyrus and right hippocampus. These findings confirmed alteration and disruption of the regional brain activity in mood regulation network in the DPD group (Sheng et al., 2014).

Salience network (SN) includes brain regions whose cortical hubs are the anterior cingulate and ventral anterior insular cortices. This network coactivates in response to various experimental tasks and conditions, suggesting a domain‐general function (Seeley, 2019). A rs‐fMRI study including 17 DPD patients, 17 ND PD patients, and 17 HC subjects found that damaged insula networks between the SN and ECN in PD might lead to DPD (Huang et al., 2020). As one of the critical nodes in the STM, the left supramarginal gyrus involved in keeping an abstract representation from the serial order information, and independently from all the content, which instead is stored separately (Guidali et al., 2019). In our study, when DPD was compared with NDPD and HC, disturbed STM regional brain network was identified and might demonstrate the attention deficit.

Our findings also selected several mALFF and ReHo features including the mALFFs of left precentral gyrus and left planum polare, and the mReHo of left middle temporal gyrus for the group of DPD versus HC. When comparing NDPD and DPD, the mALFF of left subcallosal cortex was selected. A rs‐fMRI study found abnormal baseline brain activity in the dorsolateral prefrontal cortex, the rostral anterior cingulated cortex, and the ventromedial prefrontal cortex that were positively correlated with the HAMD score. The results of abnormal ALFF values in these brain regions implied that the prefrontal‐limbic network might be associated with abnormal activities in PD patients with depression (Wen et al., 2013).

In our study, disturbed VMHC was found in the right temporal fusiform cortex, posterior division when comparing NDPD to HC. Indeed, the impaired functional connectivity within the homotopic brain regions of PD extended previous studies that the disconnection of corticostriatal circuit provided new evidence of disturbed interhemispheric connections in PD (Luo et al., 2015). A VMHC study using the seed‐based method discovered decreased VMHC values in the bilateral paracentral lobule and medial frontal gyrus in DPD compared with NDPD (Liao et al.,^2020^). A structural brain network study showed the global efficiency and characteristic path length were impaired in DPD, which indicated the topological property can be used as a potential objective neuroimaging index for early diagnosis of DPD (Gou et al.,^2018^).

However, our approach failed to extract any significant VMHC features when comparing DPD and NDPD. This may due to the smaller sample size of the DPD group. Though we could perform data augmentation to increase the sample size, the model fitting results might be compromised by the correlated structures resulting from data augmentation. Hence, for future studies, we intend to include more subjects to diminish the threats caused by the high dimensionality and to further confirm our neurological findings. In addition, the values of radiomic features before and after antidepressant treatment will also be evaluated in the future.

In conclusion, the machine learning‐based radiomic approach proposed in this study showed that high‐order radiomic features that quantify the functional connectivity and activity of the brain can be used for the diagnosis of DPD and NDPD with high accuracy.

## CONFLICT OF INTEREST

The authors have no conflict of interest to declare.

## AUTHOR CONTRIBUTIONS

QH and WL conceived and designed the study. QH, XZ, XC, JZ, SZ, CX, and WL performed the experiments. XZ, XC, and QH wrote the manuscript. XC and QH reviewed and edited the manuscript. All authors read and approved the manuscript.

### Peer Review

The peer review history for this article is available at https://publons.com/publon/10.1002/brb3.2103.

[Correction added on March 20, 2021, after first online publication: Peer review history statement has been added.]

## Supporting information

Supplementary Material

Supplementary Material

## Data Availability

The data that support the findings of this study are available from the corresponding author upon reasonable request. The data are not publicly available due to privacy or ethical restrictions.
