# Supplementary material for: Aberrant functional connectivity and activity in Parkinson’s disease and comorbidity with depression based on radiomic analysis
Source: Brain Behav. 2021 Mar 10;11(5):e02103. doi: 10.1002/brb3.2103 (PMC8119873; doi:10.1002/brb3.2103)
Supplement: Supplementary file 1 — Supplementary Material [file BRB3-11-e02103-s002.docx]

***SI Methods-1***

**Participants**

This study was approved by the Medical Research Ethical Committee of Nanjing Brain Hospital (Nanjing, China) in accordance with the Declaration of Helsinki, and written informed consent was obtained from all subjects. Twenty-one depressed PD (DPD) patients, 49 non-depressed PD (NDPD) patients and 50 matched healthy controls (HC) were recruited. All the 21 DPD subjects did not receive antidepressant treatment before recruitment. All the demographic characteristics and clinical symptom ratings were collected before MRI scanning, and all patients were in the ON state during the MRI scan.

All subjects underwent a complete neurological and psychological status assess, and a review of medical history records. Mini-mental state examination (MMSE) was used to evaluate cognition. The severity of depression was quantified using the Hamilton Depression Scale (HAMD). The neurocognitive tests were administered to each participant individually by a professional appraiser in the neuropsychological research center. All HC participants were interviewed to rule out the presence including current or past psychiatric illness, history of psychiatric illness in first-degree relatives and/or current or past significant medical or neurological illness.

The demographic and clinical data of patients with DPD, NDPD and HC were compared using a Fisher’s exact test (for sex), analysis of variance (ANOVA) (for age, education, MMSE and HAMD). The level of significance was set at p < 0.05.

**Image data acquisition**

Functional imaging data were collected transversely by using a gradient-recalled echo-planar imaging (GRE-EPI) pulse sequence with the following configurations: TR/TE = 200 ms/30 ms, flip angle = 90°, matrix = 64 × 64, FOV = 220 mm × 220 mm, thickness/ gap = 3.5 mm/0.6 mm, in-plane resolution = 3.4 mm × 3.4 mm, slices = 31. For each subject, a total of 140 volumes were obtained, resulting in a total scan time of 280s. High resolution anatomical images were acquired using a T1 fluid attenuated inversion recovery (FLAIR) sequence (TR/TE = 2530 ms/3.34 ms, flip angle = 7°, matrix = 256 × 192, FOV = 256 mm × 256 mm, slice thickness/gap = 1.33 mm/0.5 mm, 128 slices covered the whole brain). The subjects were instructed to keep their eyes closed, relax their minds and remain as motionless as possible during the data acquisition. Rubber earplugs were used to reduce noise, and foam cushioning was used to fix the head to reduce motion artifacts. The MR images were retrieved from the archive by two experienced neuroradiologists (Qingling Huang & Chen Xue).

**Data preprocessing**

The following steps were applied to the image data. For each subject, we first discarded the first 5 time points for signal equilibrium when subject was still adapting to the scanning noise. The remaining 135 images underwent slice-timing correction using the middle slice as the reference frame and head motion correction by regressing out 6 head motion signals (displacement on *x*, *y*, and *z* direction and 3 angular motion). Four subjects with more than 2.5 mm maximum displacement in any of the three dimensions or 2.5° of any angular motion were removed. Next, T1-weighted structural images of each subject were coregistrated to the resulting functional images followed by images being segmented into grey matter (GM), white matter, and cerebrospinal fluid using a new segment and DARTEL segmentation algorithm. The functional images were spatially normalized to the Montreal Neurological Institute (MNI) space with 3mm×3mm×3mm cubic voxels. After normalization, images were spatially smoothed with a 4 mm full width at half maximum (FWHM) Gaussian kernel and detrended using linear, quadratic or higher order polynomial algorithms. Note that when calculating Regional Homogeneity (ReHo), the smoothing procedure was omitted for maintaining the measuring accuracy. After smoothing and detrending, we further regressed out nuisance covariates including the Friston 24 motion parameters (Friston, Williams et al. 1996), white matter, global signals and cerebrospinal fluid signals and applied temporal filter (0.01 Hz < f < 0.08 Hz) to diminish high-frequency noise.
