# Supplementary material for: Aberrant functional connectivity and activity in Parkinson’s disease and comorbidity with depression based on radiomic analysis
Source: Brain Behav. 2021 Mar 10;11(5):e02103. doi: 10.1002/brb3.2103 (PMC8119873; doi:10.1002/brb3.2103)
Supplement: Supplementary file 2 — Supplementary Material [file BRB3-11-e02103-s001.docx]

**2. Materials and Methods** (Clinical data and data preprocessing procedure has all been applied in our published issue, Cao, Wang et al.,[Front Neurosci](https://www.ncbi.nlm.nih.gov/pmc/articles/PMC7373781/). 2020; 14: 751. doi: [10.3389/fnins.2020.00751](https://dx.doi.org/10.3389/fnins.2020.00751))

**2.1 Participates and clinical evaluation**

Seventy PD patients including 21 PD patients with depression (DPD), 49 PD patients without comorbidity of depression (NDPD) were recruited along with 50 matched healthy controls (HC). The details regarding the diagnostic criteria and clinical evaluation of NDPD and DPD groups are provided in ***SI Methods-1.***

**2.2 Image data acquisition**

Image data collected using a Siemens Verio 3.0-Tesla signal scanner (Siemens, Germany) in the department of radiology of Nanjing Brain Hospital. The details regarding image acquisition parameters (structure images and rsfMRI images) are provided in ***SI Methods-1.***

**2.3 Data preprocessing**

Image data was preprocessed using Data Processing Assistant for Resting-State fMRI (<http://rfmri.org/DPARSF>) based on Statistical Parametric Mapping (SPM12, http:// [www.fil.ion.ucl.ac.uk/spm/](http://www.fil.ion.ucl.ac.uk/spm/)) operated on the Matlab platform. The details regarding image preprocessing are provided in ***SI Methods-1.***

**2.4 Image feature extraction**

**2.4.1 ReHo analysis**

The method of Regional Homogeneity (ReHo) (Zang, Jiang et al. 2004) was used to analyze characteristics of regional brain neural activity of the temporal homogeneity. During the processing procedure, the ReHo magnitudes may be influenced by some preprocessing methods such as spatial smoothing R-fMRI time series(Zuo, Xu et al. 2012). In order to get rid of this potential issue, preprocessed rs-fMRI data was used for calculating ReHo without the spatial smoothing step. All individual ReHo maps were computed with a 4 mm FWHM Gaussian kernel for spatially smoothing. In particular, we divided the mean ReHo of the whole brain within each voxel in the ReHo map to obtained mReHo maps. We further segmented the mReHo maps and extract all the 112 ROI signals based on the Harvard-Oxford atlas (HOA) using the open Resting-State fMRI Data Analysis Toolkit, REST (<http://restfmri.net/forum/index.php?q=rest>).

**2.4.2 ALFF and VHMC extraction**

Slow fluctuations in brain activity are fundamental features of the resting state for determining correlated activity between resting state networks and brain regions. The relative magnitude of these slow fluctuations can discriminate between subjects and brain regions. Amplitude of Low Frequency Fluctuations (ALFF) (Zang, He et al. 2006) are related measures that quantify the amplitude of these low frequency oscillations. We calculated individual ALFF maps within the frequency range between 0.01 and 0.1 Hz, and the mALFF maps by dividing the mean ALFF of the whole brain within each voxel in the ALFF maps. Using the HOA, we ended up with 112 mALFF values after extracting the ROI signals based on the mALFF maps.

Voxel-Mirrored Homotopic Connectivity (VMHC) quantifies functional homotopy by providing a voxel-wise measure of connectivity between hemispheres. VMHC calculates the connectivity between each voxel in one hemisphere and its mirrored counterpart in the other (Zuo, Kelly et al. 2010). By segmenting the VMHC maps via HOA, we also got 112 VHMC values.

**2.4.3 RSFC extraction**

Resting-state functional connectivity (RSFC) analysis can effectively estimate spontaneous functional activity and measure the temporal correlation within spatially remote neurophysiological events. The preprocessed rs-fMRI images were all segmented into 112 ROIs based on HOA. The rs-fMRI time courses of all the voxels within each ROI were averaged, the mean time series of each ROI were acquired. Pearson’s correlation analysis was conducted on each pair of ROI time series (i.e. 112 × 111/2 = 6216 pairs in total). The 6216 correlation coefficients were then transformed into z-scores by Fisher’s z transformation and retained as the RSFC metrics.

**2.5 feature selection and model validation**

The candidate features cover all the aforementioned metrics including ReHo, mALFF, VHMC, RSFC and the clinical data. To build our model, we first randomly split the dataset into training set and testing set for three binary classifications: DPD vs HC, NDPD vs HC, DPD vs NDPD. Each classification included three steps: (i) training, (ii) validation, and (iii) testing. First, MRI data of each classification dataset was randomly split into a large training and validation set (70% of images) and a testing set (30% of images), while maintaining the respective ratio. All steps of feature selecting and model training were only performed and based on the training set.

Our ultimate goal was to identify the most discriminative variables for each classification. However, as we had a comparably smaller sample size and a total of 6557 features, the dimension reduction was need to improve the accuracy in the following step of building the model for classification(Wang, Sun et al. 2019). Hence, we adopted the least absolute shrinkage and selection operator (Lasso) method that assigns a penalty to the coefficients and eliminates variables with zero coefficient value. 10-fold cross validation was used to get the optimal penalty parameter for Lasso, and the features with nonzero regression coefficients were retained. The methods of Lasso prediction, support vector machine (SVM) and random forest were carried out for classifying subjects based on the selected features. The Lasso fitting and prediction were implemented through the glmnet and predict function, respectively. and visualized according to different metrics including the area under curve (AUC), receiver-operating characteristic (ROC) curve, accuracy, true positive rate, and false positive rate were compared for the two different machine learning methods in the training and test sets. The statistical analysis was conducted with R 3.5.0. The flowchart of this study is shown in Figure 1.


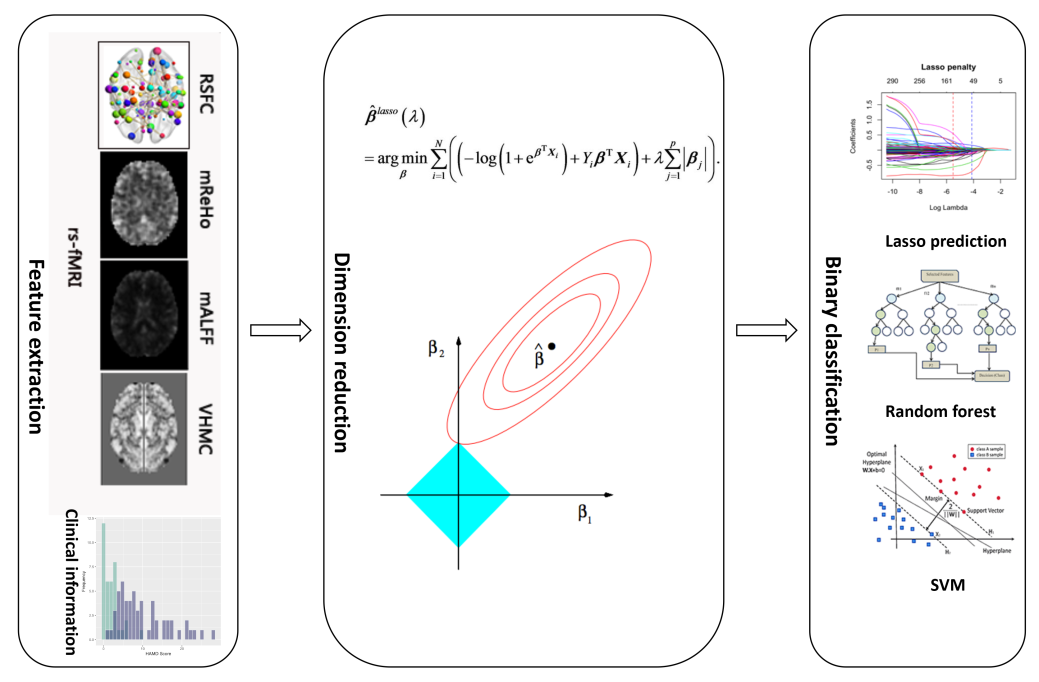


Figure 1. Flowchart of the study. we extracted the 6557 metrics after the rs-fMRI images preprocessed. Then, Lasso regression was carried out to reduce the number of features. Last, Lasso prediction, random forest and SVM were used to differentiate between different categories of subjects.
